# *Acetobacter indonesiensis* Pneumonia after Lung Transplant

**DOI:** 10.3201/eid1406.071236

**Published:** 2008-06

**Authors:** Fadi Bittar, Martine Reynaud-Gaubert, Pascal Thomas, Stéphanie Boniface, Didier Raoult, Jean-Marc Rolain

**Affiliations:** * Unité de Research sur les Maladies Infectieuses et Tropicales Emergentes, Marseille, France; † Hôpital Sainte Marguerite, Marseille

**Keywords:** Actobacter indonesiensis, cystic fibrosis, colistin resistance, lung transplant, pneumonia, letter

**To the Editor:** Unusual and multiresistant bacterial infections are increasingly reported in cystic fibrosis (CF) patients (*1*). On January 25, 2007, a 31-year-old man with CF (mutation ΔF 508 and I 507) was admitted to our institution in Marseille, France, for lung transplantation. His immunosuppressive regimen included IV cyclosporin A (for the first 6 days with conversion to oral tacrolimus thereafter), azathioprine, and corticosteroids. Induction therapy that used antithymocyte globulin was administered for the first 3 days (Thymoglobuline, Genzyme Corporation, Naarden, the Netherlands). Since 2003, the patient was chronically colonized by methicillin-resistant *Staphylococcus aureus* (MRSA), *Pseudomonas aeruginosa* (susceptible only to colistin sulfomethate), and *Candida albicans.* Preemptive treatment with antimicrobial agents including colistin sulfomethate, tobramycin sulfate, ceftazidime, and linezolid was administered, starting on posttransplant day 1; prophylactic caspofungin, followed by inhaled amphotericin B, was given for the first month. Six and 9 days, respectively, after surgery, sputa from the patient showed *P. aeruginosa* and MRSA.

On postoperative day 11, the patient’s clinical condition worsened. Leukocytes increased to 13.84 × 10^9^/L. In addition to *P. aeruginosa* (10^4^ CFU/mL) and MRSA (10^3^ CFU/mL), culture of later sputum samples yielded the growth of 10^4^ CFU/mL of gram-negative, catalase-positive, and oxidase-negative bacillus (isolate 7120034) on CEPACIA agar (AES, Combourg, France) after 72 hours of incubation at 30°C. API 20NE, API 20E, and VITEK 2 Auto system (bioMérieux, Marcy l’Etoile, France) did not identify the bacillus. This bacterium was multiresistant to antimicrobial agents, including colistin, and was susceptible only to imipenem, rifampin, and aminoglycosides. The final identification of this isolate as *Acetobacter indonesiensis* was achieved after partial sequencing of 16S rRNA gene, as previously described (*2*) (GenBank accession no. AJ199841, 99% similarity). The sequence of our isolate has been deposited in GenBank under the accession no. EF681860. The phylogenetic position of isolate 7120034 among other gram-negative bacteria is shown in the Figure.

**Figure:**
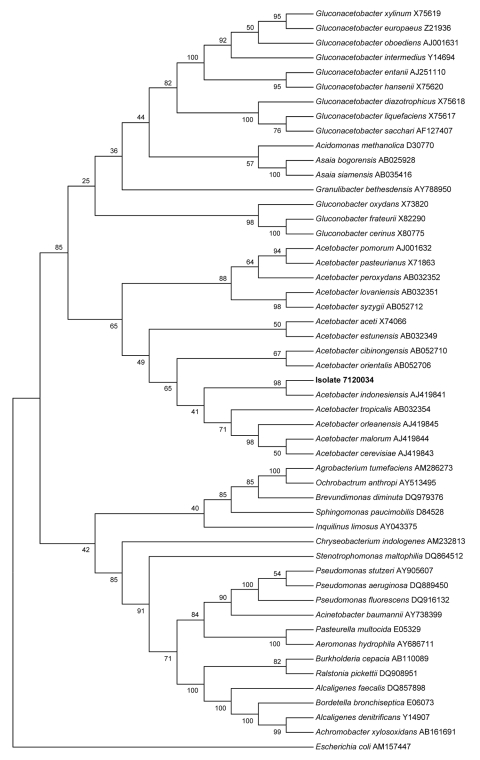
Phylogenetic tree showing the position of *Acetobacter indonesiensis* (isolate 7120034, GenBank accession no. EF681860), in **boldface**, within acetic acid bacteria and other gram-negative rods. The tree was based on 16S rDNA comparison by the neighbor-joining method. Numbers along the branches indicate bootstrap values.

Tobramycin was stopped at day 11, colistin and ceftazidime were stopped at postoperative day 14, linezolid was maintained for 10 additional days, and gentamicin (for 13 days) was added to the patient’s drug therapy on February 10. Despite this treatment, *A. indonesiensis* was cultured from sputa obtained on February 16 (10^4^ CFU/mL) and February 20 (10^2^ CFU/mL), respectively. Six days later, leukocytes decreased to 8.62 × 10^9^/L, and the patient’s condition improved. He was discharged at the beginning of March. During 7 months of follow-up, the *A. indonesiensis* strain was not found again.

Acetic acid bacteria are gram-negative bacilli classified into the genera *Acetobacter, Gluconobacter, Gluconacetobacter, Acidomonas,* and the recently described genus *Asaia*; these bacteria belong to the α subgroup of *Proteobacteria* (*3*). Isolates of this family are recognized as food-associated bacteria and are able to grow at acidic pH (*4*). Three species have been reported as emerging pathogens in humans: *Asaia bogorensis* (in a case of peritonitis in a patient with a peritoneal dialysis catheter [*5*]); *Granulibacter bethesdensis* (in 3 cases of lymphadenitis associated with chronic granulomatous disease [*6*]); and *Acetobacter cibinongensis* (a recent case of bacteremia in a patient receiving chronic hemodialysis for end-stage renal failure [*7*]).

*A. indonesiensis* has been isolated from fruits and flowers in Indonesia (*8*); only 3 reports on it have been published (*3*,*8*,*9*). In our patient, we believe that this multidrug-resistant bacterium was the primary cause of the infection because the patient was eventually cured after an adapted antimicrobial drug therapy. Because this bacterium grows easily at acidic pH (*4*), a classic condition in the CF airway surface liquid, acidity might contribute to bacterial adhesion and colonization (*10*). Because acetic acid bacteria have never been isolated from human flora, the source of the contamination for our patient remains unknown.

We also report the antimicrobial drug susceptibility of this bacterium. It was multiresistant, especially to colistin. Antimicrobial drug susceptibility results were obtained by using Vitek 2 Auto system because of the absence of growth on Mueller-Hinton agar. This pattern was also the case for *G. bethesdensis* (*6*). The antimicrobial susceptibility of *A. cibinongensis* could not be validated because of the lack of interassay reproducibility (*7*). Initial antimicrobial drug therapy for this patient with amoxicillin, pristinamycin, and cefazolin did not cure the patient; he was eventually cured after the therapy was switched to tobramycin (*7*).

Our findings reemphasize the emergence of new colistin-resistant pathogens in CF patients, as recently reported for *Inquilinus limosus* (*1*). The increased clinical use of nebulized colistin for *P. aeruginosa* infection in CF patients may select specific colistin-resistant bacteria in such populations. In summary, this report of a respiratory tract infection caused by *A. indonesiensis* after lung transplantation in a French CF patient supports that this multidrug-resistant bacterium may be an emerging opportunistic pathogen in immunocompromized patients, including CF patients with a lung transplant.
